# Protective and risk factors of impaired awareness of hypoglycemia in patients with type 1 diabetes: a cross-sectional analysis of baseline data from the PR-IAH study

**DOI:** 10.1186/s13098-023-01024-x

**Published:** 2023-04-25

**Authors:** Naoki Sakane, Ken Kato, Sonyun Hata, Erika Nishimura, Rika Araki, Kunichi kouyama, Masako Hatao, Yuka Matoba, Yuichi Matsushita, Masayuki Domichi, Akiko Suganuma, Seiko Sakane, Takashi Murata, Fei Ling Wu

**Affiliations:** 1 Division of Preventive Medicine, Clinical Research Institute, National Hospital Organization Kyoto Medical Center, 1-1 Mukaihata-cho, Fukakusa, Fushimi-ku, 612-8555 Kyoto, Japan; 2 Diabetes center, National Hospital Organization Osaka National Hospital, 2-1-14 Hoenzaka, Chuo-ku, 540-0006 Osaka, Japan; 3 Department of Diabetes and Endocrinology, National Hospital Organization Mie National Hospital, 357 Ozatokubota-cho, 514-0125 Tsu, Mie, Japan; 4 Department of Diabetes and Metabolism, National Hospital Organization Hyogo-Chuo National Hospital, 1314Ohara, 669-1515 Sanda, Hyogo Japan; 5 Department of Diabetes and Endocrinology, National Hospital Organization Himeji Medical Center, 68 Honmachi, 670-0012 Himeji, Hyogo Japan; 6 Department of Diabetes, Endocrinology and Metabolism, National Hospital Organization Kokura Medical Center, 10-1 Harugaoka, Kitakyushu Kokuraminami-ku, 802-0803 Fukuoka, Japan; 7 Department of Diabetology and Metabolism, National Hospital Organization Okayama Medical Center, 1711-1 Tamasu, Okayama Kita-ku, 701-1192 Okayama, Japan; 8 Department of Clinical Nutrition, National Hospital Organization Kyoto Medical Center, 1-1 Mukaihata-cho, Fukakusa, Fushimi-ku, 612-8555 Kyoto, Japan; 9 Diabetes Center, National Hospital Organization Kyoto Medical Center, 1-1 Mukaihata-cho, Fukakusa, Fushimi-ku, 612-8555 Kyoto, Japan; 10 Department of Nursing, Chang Gung University of Science and Technology, No. 261, Wenhua 1st Rd, Guishan District, 333 Taoyuan City, Taiwan

**Keywords:** Impaired hypoglycemia awareness, Type 1 diabetes, Continuous subcutaneous insulin infusion

## Abstract

**Background:**

Hypoglycemia in type 1 diabetes (T1D) is associated with mortality and morbidity, especially when awareness of hypoglycemia is impaired. This study aimed to investigate the protective and risk factors for impaired awareness of hypoglycemia (IAH) in adults with T1D.

**Methods:**

This cross-sectional study enrolled 288 adults with T1D (mean age, 50.4 ± 14.6 years; male, 36.5%; diabetes duration, 17.6 ± 11.2 years; mean HbA1c level, 7.7 ± 0.9%), who were divided into IAH and non-IAH (control) groups. A survey was conducted to assess hypoglycemia awareness using the Clarke questionnaire. Diabetes histories, complications, fear of hypoglycemia, diabetes distress, hypoglycemia problem-solving abilities, and treatment data were collected.

**Results:**

The prevalence of IAH was 19.1%. Diabetic peripheral neuropathy was associated with an increased risk of IAH (odds ratio [OR] 2.63; 95% confidence interval [CI] 1.13–5.91; P = 0.014), while treatment with continuous subcutaneous insulin infusion and hypoglycemia problem-solving perception scores were associated with a decreased risk of IAH (OR, 0.48; 95% CI, 0.22–0.96; P = 0.030; and OR, 0.54; 95% CI, 0.37–0.78; P = 0.001, respectively). There was no difference in continuous glucose monitoring use between the groups.

**Conclusion:**

We identified protective factors in addition to risk factors for IAH in adults with T1D. This information may help manage problematic hypoglycemia.

**Trial registration:**

University hospital Medical Information Network (UMIN) Center: UMIN000039475). Approval date 13 February 2020.

## Background

Adults with type 1 diabetes mellitus (T1D) and impaired awareness of hypoglycemia (IAH) have a reduced ability to perceive hypoglycemic symptoms and are at risk of severe hypoglycemic events because of less than immediate appropriate corrective therapy [1]. Autonomic symptoms are typically lost before general malaise and neuroglycopenic symptoms. Therefore, individuals with IAH may plan to loosen tight glucose management and intentionally omit insulin injection to prevent severe hypoglycemia (SH). In individuals with T1D, IAH is highly prevalent with or without continuous glucose monitoring (CGM) [2]. Although recurrent hypoglycemia [3], diabetic neuropathy [4], longer diabetes duration [5], genetic factors [6], and personality traits of alexithymia and perfectionism [7] are candidate risk factors for IAH, their pathogenesis is still unclear. In individuals with IAH, counter-regulatory responses are blunted to subsequent hypoglycemic episodes due to recurrent mild to moderate hypoglycemia. This is called hypoglycemia-associated autonomic failure, which is a cellular adaptation [8]. It is also unclear which lifestyle factors are associated with the risk of IAH, although antecedent exercise, excessive drinking, psychological stress, and sleep disturbance may induce recurrent hypoglycemia [9, 10]. In contrast, diabetes treatment technologies (e.g. CGM and continuous subcutaneous insulin infusion (CSII)), and hypoglycemia-solving abilities might affect IAH status. Therefore, this study aimed to investigate the protective and risk factors of IAH in adults with T1D.

## Materials and methods

This is an exploratory and cross-sectional study, using the STROBE instrument included in reports of cross-sectional studies. The study was approved by the National Hospital Organization (NHO) Central Research Ethics Committee (R2-0117002). Participants and Settings Between February 2020 and March 2022, we enrolled adults with IAH at seven NHO collaborator center in Japan. The inclusion criteria were type 1 diabetes, diabetes duration ≥ 1 year, age ≥ 20 years, and attending a collaborating center. The exclusion criteria were non-insulin therapy, anti-dementia drug use, and inappropriate cases (i.e. difficulties to answer the survey) judged by the research director or coordinators.

## Diabetic complications

Treatment of diabetic retinopathy, nephropathy, and peripheral neuropathy was performed by certified diabetologists according to the treatment guidelines for diabetes 2018–2019.

Diabetic retinopathy was assessed by an ophthalmologist using retinal photography. Retinopathy was classified as absent, simple, preproliferative, and proliferative. Diabetic nephropathy was classified as stage 1 to 5 based on the estimated glomerular filtration rate and albuminuria or hemodialysis stage [11]. Diabetic peripheral neuropathy (DPN) was considered present following the criteria after patients were diagnosed with diabetes and excluded polyneuropathy, except diabetic polyneuropathy. DPN was determined to be positive in the presence of ≥ two of the three criteria: (1) subjective neurological symptoms (pain, dysesthesia, or numbness in the bilateral lower extremities); (2) decreased or absent bilateral Achilles tendon reflexes; and (3) diminished bilateral vibratory sensation (hypesthesia) at the malleolus medialis (< 10 s at 128 Hz using a tuning fork) [12]. Coefficient of variation of R-R intervals (CV-RR) was calculated automatically by a computed analyzer that collected 100 R-R intervals and divided the standard deviation by the mean value. CV-RR < 3% was indicative of diabetic cardiac autonomic neuropathy (DCAN) [13]. The mean QTc interval was calculated using Bazett’s formula, and a QTc > 440 ms was considered prolonged [14].Hemoglobin A1c (HbA1c) level, glycated hemoglobin (GA) level, levels of liver enzymes, and lipid profiles were collected from the medical records. Furthermore, the GA/HbA1c ratio, which reflects glucose variability, was calculated by dividing the GA level by the HbA1c level [15].

## Impaired awareness of hypoglycemia and hypoglycemic symptoms

The IAH was determined using the Clarke method [16] and a score ≥ 4 implies IAH. Hypoglycemic symptoms were evaluated using the Edinburgh hypoglycemia scale [17, 18]. Self-reported number of SH episodes, defined as “hypoglycemia that you were unable to treat yourself,” in the preceding year was also collected.

## Diabetes-related distress and hypoglycemia problem-solving abilities

Distress related diabetes management was assessed using the PAID questionnaire and high score of ≥ 40 points indicates severe distress [19, 20]. The Hypoglycemia Fear Survey (HFS) were used to have a fear of hypoglycemia [21, 22]. The general utility index was calculated using the EuroQoL 5-dimension (EQ-5D) [23, 24]. The hypoglycemia problem-solving scale (HPSS), which has 24 items and seven subscales, was used for hypoglycemia problem-solving ability [25].

## Lifestyle factors

Self-administered questionnaires regarding lifestyle factors (exercise, dietary habits, drinking, smoking, and sleep habits) were collected [26], and sleep debt [27] and healthy lifestyle score [28] were calculated.

## Sample size

The prevalence of IAH is approximately 20% [29]. Therefore, a minimum sample size of 200 completers (40 participants with IAH and 160 participants without IAH) was needed to achieve an appropriate significance level of 5% and power of 0.8, if the prevalence of IAH of 20% and effect size of 0.5 (medium) were estimated.

## Data analyses

Qualitative variables were compared using Fisher’s exact test. Quantitative variables were compared using the t-test or the Mann–Whitney U test. Logistic regression was performed to estimate the odds ratio (OR) with 95% confidence interval (CI). Cronbach’s alpha was calculated to assess the internal consistency. Correlation coefficients (Spearman’s rho, ρ) between the HPSS and psychological distress scales was examined using Spearman’s rank correlation. All P-values < 0.05 indicated significant dependencies. The analysis was conducted using R program version 4.1.2.

## Results

### Participants

The study was conducted on 288 adults with T1D and IAH (mean age, 50.4 ± 14.6 years; male, 36.5%; diabetes duration, 17.6 ± 11.2 years; mean HbA1c level, 7.7 ± 0.9%), who were divided into control and IAH groups.

### Diabetic complications, treatment, lifestyle factors, and laboratory data

DPN was more prevalent in the IAH group than in the control group. Moreover, the prescription rate of mecobalamin was higher in the IAH group than that in the control group. There was no difference in HbA1c levels and other complications, except DPN, between the groups. Treatment with CSII was less prevalent in the IAH group than in the control group, but there was no difference in CGM usage between the groups (Table 1). There was no difference in healthy lifestyle score, sleep debt, and excessive drinking rate between the groups (Table 2). There was no difference in laboratory data, including HbA1c level and GA/HbA1c ratio, between the groups (Table 3).

**Table 1:** Baseline characteristics of the study participants

Variables	Control group (n = 233)	IAH group (n = 55)	P-value
Age, years Male sex, % Diabetes duration, years BMI, kg/m^2^ HbA1c, %	49.9 (14.5) 36.5 17.6 (11.0) 23.4 (3.5) 7.7 (0.9)	52.8 (15.1) 36.4 17.6 (11.8) 22.9 (4.4) 7.5 (1.0)	0.179 > 0.999 0.998 0.355 0.130
Diabetic complication			
Retinopathy, %			
NDR/SDR/PPDR/PDR Photocoagulation Nephropathy, %	75.0/16.1/4.9/4.0 10.3	79.6/12.2/2.0/6.1 12.7	0.681 0.629
1st/2nd/3rd/4th/5th stage	81.0/13.4/3.4/0.4/1.7	85.2/14.8/0/0/0	0.673
Peripheral neuropathy, % Severe hypoglycemia, %	12.0 3.4	26.5 30.9	0.014* < 0.001*
Treatment			
CSII, % CGM usage, % isCGM, % rtCGM, % TDD/BW, U/kg Antihypertensive drug, % Cholesterol-lowering drug, % Mecobalamin, %	39.5 56.2 32.2 24.0 0.6 (0.2) 23.2 26.6 2.6	23.6 56.4 36.4 20.0 0.6 (0.2) 20.0 25.5 9.1	0.030* > 0.999 0.633 0.597 0.090 0.721 > 0.999 0.039*
ECG			
QTc (Bazett’s formula), ms > 440 ms, % CV-RR, % < 3%, %	416.4 (27.8) 14.3 3.5 (1.7) 48.6	414.2 (23.1) 14.3 3.1 (1.4) 50.0	0.697 > 0.999 0.344 > 0.999

Mean(SD) or  %. *P-value < 0.05

BMI, body mass index; HbA1c, glycated hemoglobin A1c; NDR, no diabetic retinopathy; SDR, simple diabetic retinopathy; PPDR, pre-proliferative diabetic retinopathy; PDR, proliferative diabetic retinopathy; CSII, continuous subcutaneous insulin infusion; isCGM, intermittently scanned continuous glucose monitoring; rtCGM, real-time CGM; TDD, total daily dose; BW, body weight; ECG, electrocardiogram; QTc, corrected QT interval; CV-RR, coefficient of variance of the heart rate variation

**Table 2:** Lifestyle factors in adults with T1D with or without IAH

Variables	Control group	IAH group	P-value
Lifestyle			
Skipping breakfast, % Fast eating, % Late-night dinner eating, % Snack and sweetened beverage, % Fruits ≥ 1 intake per day, % Milk ≥ 1 intake per day, % Fish ≥ 1 intake per day, % Vegetable ≥ 5 dishes intake per day, % Exercise habit, % Physical activity, % Fast walking, % Overwork, % Current smoking, % Drinking everyday, % Excessive drinking, % Healthy lifestyle score, points	10.0 33.3 26.0 74.6 27.3 57.6 6.9 3.9 31.2 54.5 44.6 20.8 17.3 15.6 9.1 4.3 (1.4)	9.1 40.0 29.1 69.1 18.2 54.5 10.9 7.3 34.5 53.7 52.7 18.5 21.8 21.8 10.9 4.2 (1.5)	> 0.999 0.349 0.615 0.400 0.228 0.762 0.395 0.287 0.632 > 0.999 0.295 0.852 0.440 0.315 0.617 0.392
Sleep			
Average sleep time, min Sleep time on a weekday, min Sleep time on a weekend, min Sleep debt, min Nonrestorative sleep, %	395 (58) 380 (61) 433 (79) 53 (74) 39.0	391 (87) 374 (90) 432 (102) 57 (78) 40.0	0.704 0.632 0.955 0.711 0.879

Mean(SD) or %

**Table 3:** Laboratory data of the study participants

Variables	Control group	IAH group	P-value
TP, g/dL Alb, g/dL AST, U/L ALT, U/L GGT, IU/L BUN, mg/dL Serum creatinine, mg/dL eGFR HbA1c, % GA, % GA/HbA1c ratio LDL-C, mg/dL HDL-C, mg/dL TG, mg/dL	6.9 (0.4) 4.2 (0.3) 20.2 (7.4) 17.6 (11.2) 21.4 (19.1) 15.5 (5.8) 0.9 (1.0) 77.8 (22.2) 7.7 (0.9) 22.0 (3.7) 2.9 (0.3) 113.0 (27.9) 79.2 (20.1) 94.0 (54.8)	6.9 (0.4) 4.1 (0.3) 21.0 (7.4) 16.7 (8.1) 22.1 (19.2) 14.3 (4.1) 0.8 (0.5) 78.6 (14.5) 7.5 (1.0) 21.7 (3.6) 2.9 (0.4) 107.0 (23.4) 77.6 (20.0) 92.6 (73.9)	0.824 0.547 0.482 0.592 0.806 0.155 0.530 0.801 0.130 0.591 0.520 0.143 0.605 0.865

Mean (SD)

TP, total protein; Alb, albumin; ALT, alanine transaminase; AST, aspartate aminotransferase; GGT, gamma-glutamyl transferase

BUN, blood urea nitrogen; eGFR, estimated glomerular filtration rate; HbA1c, glycated hemoglobin; GA, glycated albumin

LDL-C, low-density lipoprotein cholesterol; HDL-C, high-density lipoprotein cholesterol; TG, triglyceride

DPN was associated with an increased risk of IAH (OR, 2.63; 95% CI, 1.13-5.91; P = 0.014), while treatment with CSII was associated with a decreased risk of IAH (OR, 0.48; 95% CI, 0.22-0.96; P = 0.030) (Table 4). There was no difference in CGM usage, CV-RR, and QTc intervals between the groups.

**Table 4:** Odds ratio of interest variables for IAH in adults with T1D

Variables	Odds ratio (95%CI)	P-value
Diabetic complications		
Diabetic peripheral neuropathy Retinopathy, NDR/SDR/PPDR vs. PDR Nephropathy, 1st vs. 2nd/3rd/4th/5th stage	2.63 (1.13–5.91) 1.56 (0.26–6.55) 0.74 (0.28–1.74)	0.014* 0.456 0.560
Diabetes treatment		
CGM usage CSII treatment	1.01 (0.53–1.91) 0.48 (0.22–0.96)	>0.999 0.030*
Hypoglycemia problem-solving scale		
1. Problem-solving perception 2. Detection control 3. Identifying problem attributes 4. Setting problem-solving goals 5. Seeking preventive strategies 6. Evaluating strategies 7. Immediate management	0.54 (0.37–0.78) 0.95 (0.75–1.21) 1.09 (0.84–1.42) 1.00 (0.77–1.32) 0.98 (0.72–1.33) 0.86 (0.63–1.17) 1.12 (0.83–1.51)	0.001* 0.690 0.503 0.973 0.886 0.339 0.446

Odds ratio (95% CI)。*P-value < 0.05

### Hypoglycemic symptoms, diabetes distress, and hypoglycemia problem-solving abilities

The mean autonomic symptom scores, except for palpitations and hunger in the IAH group compared to the control group, were significantly reduced, while the mean neuroglycopenic symptom scores were relatively lower in the control group than in the IAH group. The scores are shown in (Table 5). The average PAID and HFS-worry scores in the IAH group were significantly higher than those in the control group, and there was no difference in the PHQ-9 and HFS-behavior scores between the groups. The HPSS, composed of 24 items, demonstrated high internal consistency, as reflected by a Cronbach’s alpha coefficient of 0.883. A weak positive correlation of the HPSS score with HFS-B (ρ = 0.331, P<0.001) and HFS-W (ρ = 0.162, P = 0.006) were observed, although no significant correlation of the HPSS with age, HbA1c, PAID and PHQ-9 scores were observed. Hypoglycemia problemsolving perception score of HPSS was associated with a decreased risk of IAH (OR, 0.54; 95% CI, 0.37–0.78; P = 0.001), although there was no difference in the other six subscales between groups.

**Table 5:** Hypoglycemic symptoms in adults with T1D with or without IAH

Variables	Control group	IAH group	P-value
Autonomic, points			
Sweating Palpitations Shaking Hunger	3.6 (1.9) 3.5 (1.8) 3.7 (1.8) 3.6 (1.9)	2.9 (1.9) 3.1 (1.9) 3.1 (1.8) 3.1 (1.8)	0.019* 0.122 0.013* 0.087
Neuroglycopenic, points			
Confusion Drowsiness Odd behavior Speech difficulty Incoordination	1.9 (1.5) 2.1 (1.5) 1.5 (1.1) 1.7 (1.3) 2.5 (1.7)	2.5 (2.0) 2.7 (1.8) 2.1 (1.6) 2.6 (2.0) 3.1 (1.7)	0.016* 0.014* 0.003* < 0.001* 0.010*
General malaise, points			
Headache Nausea Total score, points	1.8 (1.4) 1.6 (1.1) 26.8 (10.1)	2.1 (1.7) 1.9 (1.6) 28.4(12.5)	0.150 0.059 0.328
Psychological			
Paid, points PHQ-9, points HFS-B, points HFS-W, points EQ-5D utility index	29.1 (19.4) 3.9 (4.2) 18.0 (6.3) 10.0 (9.1) 0.91 (0.13)	35.7 (18.4) 4.9 (4.1) 18.2 (6.8) 14.9 (10.5) 0.86 (0.17)	0.022* 0.113 0.798 0.001* 0.015*

Mean (SD). *P-value<0.05

## Discussion

This is the first study to identify protective factors (treatment with CSII and higher problem-solving perception) and risk factors (DPN) of IAH in Japanese adults with T1D.

## DPN and IAH

Cross-sectional and observational studies have indicated that DPN, cardiac autonomic neuropathy, and gastroparesis are associated with SH [30, 32–34, 31]. However, Olsen et al. reported that IAH was not associated with autonomic dysfunction or DPN in adults with T1D[35]. Conversely, Flatt et al. reported that peripheral neuropathy was more prevalent in patients with SH than in patients without SH in the 24-month follow-up of the HypoCOMPASS study (39% vs. 4.7%, respectively) [36]. This discrepancy between the results is unknown. The diagnostic criteria, ethnicity, and differences in the population with diabetes may explain this. Furthermore, the mechanism by which DPN causes IAH remains unclear. CV-RR and abnormality of the QTc interval were not associated with severe hypoglycemic attacks in this study although SH attacks were independently associated with a prolonged QTc interval in 3,248 patients with T1D from the EURODIAB IDDM Complications Study [34]. DPN is associated with cognitive impairments in adults with T1D [37]. In this study, we observed deficits in autonomic symptoms in adults with T1D and IAH. Cognitive impairment may affect the perception of hypoglycemia. In addition, repeated hypoglycemia can cause DPN as reported in animal experiments. Further large and long-standing examinations are required to confirm these issues in the future.

## Diabetes-related technologies

In this study, treatment with CSII was less prevalent in adults with T1D and IAH although CGM devices were not associated with an increased risk of IAH. New technologies, including CGM, aim to improve the awareness status of hypoglycemia. However, several studies have suggested that IAH persists even with CGM usage. Reddy et al. reported that rtCGM (Dexcom G5) more effectively reduces time spent in hypoglycemia at 8 weeks compared to isCGM (Abbott Freestyle Libre) in 40 adults with T1D and IAH using a multiple daily injections (MDI) regimen [38]. Moreover, rtCGM systems reduce unawareness of hypoglycemia in children, adolescents, and adults with T1D [39]. Further examinations including rtCGM and large samples are required to confirm these issues because the rtCGM usage rate was low in this study. Conversely, treatment with CSII may be useful in reducing unawareness of hypoglycemia in adults with T1D. The clinical statement for the management of problematic hypoglycemia (2015) recommended structured education, MDI with real-time CGM or CSII, a sensor-augmented pump with or without a low glucose suspension feature, and pancreatic islet transplantation [40]. Observational studies based on these guidelines are needed to confirm these issues in the future.

## Limitation of the study

The strength of the study includes a validated self-administered questionnaire. However, our study had some limitations. This study used a cross-sectional study design to make a causal inference. DPN was estimated as presence or absence; therefore, the severity of peripheral neuropathy was not evaluated. Even with more prescription of mecobalamin, that could be a confusing factor because some studies showed a positive contribution on the DPN symptoms [41]. Evaluating the severity of DPN would provide the information on the relation of IAH and mecobalamin treatment with DPN. DCAN was evaluated using the CV-RR in this study. DCAN is an underdiagnosed cardiovascular complication in individuals with diabetes. In an animal model, DCAN was evaluated using histology patterns and cardiac nerve densities. QTc intervals are affected by other factors, such as obesity, arteriosclerotic macroangiopathy, and autonomic [42]. Nerve conduction studies and sympathetic skin responses are reliable methods in detecting DCAN. Furthermore, definite DCAN was defined as ≥ 2 positive cardiac autonomic tests [43]. Further examination, including the definite DCAN method, is required to compare DCAN and IAH status.

## Implication for practice

We adopted the Clarke method; however, the prevalence of IAH using the Clarke method is likely to be underestimated according to the CGM data. IAH is prevalent in adults with T1D. Moreover, the diagnosis of DPN is challenging [44, 45]. Careful attention should be paid to diabetes distress and fear of hypoglycemia. We identified protective factors for IAH as treatment with CSII and problem-solving perception of HPSS. CSII improved hypoglycemia awareness in the research setting [40], while CSII use in IAH group was less prevalent in this study. The reason of low prevalence of CSII use in IAH is not clear. Personality or diabetes distress might lower the opportunity to use CSII in this group. We should consider CSII or structured education in adults with T1D and IAH [46]. Problem-solving, which is defined as a self-directed cognitive-behavioral process by which people attempt to cope with a difficult situation, is a behavioral strategy in diabetes management. They refer to a mental process that involves discovering, analyzing, and solving problems. The problem-solving perception factor explained most of the variance between the seven factors. The problem-solving perception subscale consists of four reverse items: discouraged for failure to prevent hypoglycemia, feeling depressed or angry because of difficulties in preventing hypoglycemia, worrying about how to prevent hypoglycemia but have not taken any action, and reduced self-esteem. The intervention based on the HPSP effectively improved HbA1c levels and hypoglycemia problem-solving ability in individuals with hypoglycemia [47]. We should take into account an education program with an increased problem-solving ability of adults with T1D and IAH. This study showed that HPSS scale was easy to use and help adults with T1D and IAH in their life. However, we need to build a multidisciplinary team to educate adults with T1D and IAH using HPSS.

In conclusion, we identified protective factors in addition to risk factors for IAH in Japanese adults with T1D. This information may help manage problematic hypoglycemia. IAH has a complex pathophysiology and might lead to serious and potentially lethal consequences in patients with T1D [48]. A stepwise approach using diabetes-related technologies [49] was needed to educate and treat patients with T1D and IAH in the future.

## Declarations

### Animal studies

N/A.

## List of abbreviations

CGM: continuous glucose monitoring
CI: confidence interval
CSII: continuous subcutaneous insulin infusion
DCAN: diabetic cardiac autonomic neuropathy
DPN: diabetic peripheral neuropathy
HbA1c: hemoglobin A1c
IAH: impaired awareness of hypoglycemia
isCGM: intermittently scanned continuous glucose monitoring
MDI: multiple daily injection
rtCGM: real-time continuous glucose monitoring
T1D: type 1 diabetes
